# Optimization of the Production of 1-Phenylethanol Using Enzymes from Flowers of Tea (*Camellia sinensis*) Plants

**DOI:** 10.3390/molecules22010131

**Published:** 2017-01-13

**Authors:** Fang Dong, Ying Zhou, Lanting Zeng, Naoharu Watanabe, Xinguo Su, Ziyin Yang

**Affiliations:** 1 Guangdong Food and Drug Vocational College, Longdongbei Road 321, Tianhe District, Guangzhou 510520, China; dongfangxyz@163.com; 2 Key Laboratory of South China Agricultural Plant Molecular Analysis and Genetic Improvement & Guangdong Provincial Key Laboratory of Applied Botany, South China Botanical Garden, Chinese Academy of Sciences, Xingke Road 723, Tianhe District, Guangzhou 510650, China; yzhou@scbg.ac.cn (Y.Z.); zenglanting@scbg.ac.cn (L.Z.); 3 Graduate School of Science and Technology, Shizuoka University, 3-5-1 Johoku, Naka-ku, Hamamatsu 432-8561, Japan; watanabe.naoharu@shizuoka.ac.jp

**Keywords:** aroma, *Camellia sinensis*, 1-phenylethanol, response surface methodology, tea, volatile

## Abstract

1-Phenylethanol (1PE) can be used as a fragrance in food flavoring and cosmetic industries and as an intermediate in the pharmaceutical industry. 1PE can be synthesized from acetophenone, and the cost of 1PE is higher than the cost of acetophenone. Therefore, it is important to establish an effective and low-cost approach for producing 1PE. Our previous studies found that tea (*Camellia sinensis*) flowers, which are an abundant and waste resource, contained enzymes that could transform acetophenone to 1PE. In the present study, we extracted crude enzymes from tea flowers and optimized the production conditions of 1PE using response surface methodology. The optimized conditions were an extraction pH of 7.0, a reaction pH of 5.3, a reaction temperature of 55 °C, a reaction time of 100 min, a coenzyme NADPH concentration of 3.75 μmol/mL in the reaction assay, and a substrate acetophenone concentration of 1.25 μmol/mL in the reaction assay. The results provide essential information for future industrial 1PE production using plant-derived enzymes.

## 1. Introduction

So far, more than 1700 volatiles, which contribute to approximately 1% of all plant-specialized (secondary) metabolites currently known, have been identified in more than 90 plant families [1]. From a physiological standpoint, plant volatiles are mainly involved in protective effects against biotic stress by deterring herbivores and by attracting the enemies of herbivores, plant–plant or within-plant signaling, attractants for species-specific pollinators, and protective effects against abiotic stress possibly through direct or indirect improvement in resistance to damage by reactive oxygen species [1,2,3]. On the other hand, plant volatiles possess potential economic applications including improvement in food storage and flavor, sedation, and improvement in memory [4,5]. There are also increasing reports that plant volatiles are endowed with functions of human health benefits, especially a range of biological activities, which provides the basis of plant volatiles as lead compounds for drug discovery [6]. Owing to economic applications of plant volatiles, many attempts have been made to produce plant volatiles with high purity, such as direct extraction and isolation from plant materials, production by microorganism, chemical synthesis, and enzymatic synthesis. In generally, volatiles from plant-derived resources are accepted as “natural products”. Direct extraction and isolation of volatiles from plants have a high cost, are time-consuming, and produce a low yield. Therefore, utilization of plant-derived enzymes is an alternative approach to producing “natural volatiles”.

Tea (*Camellia sinensis*) is an important crop and cultivated in more than 30 countries including China, Japan, India, and Kenya. The most utilized part of tea plants is the leaf, which is generally used to make the most widely consumed beverage aside from water. However, less attention has been paid to tea flowers. Since asexual propagation was applied to tea plants, tea flowers became a “waste resource”, that competes with tea leaves for water and nutrients. In China, over 4.0 billion kilograms of tea flowers are available annually (see the tea flowers web reference, after the reference list) [7]. In the past fifteen years, many researchers have isolated and identified functional metabolites in tea flowers, including catechins, caffeine [8,9], floratheasaponins [10,11,12], flavonol glycosides [13], polysaccharides [14], amino acids [15], and spermidine derivatives [16]. Besides non-volatile metabolites, volatile compounds have been detected in tea flowers, and volatile phenylpropanoids and benzenoids, especially 1-phenylethanol (1PE), have been identified as major volatiles [17,18,19,20]. In contrast to metabolites, enzymes in tea flowers have attracted less attention, and only proteases [21] and some enzymes involved in the formation of volatiles [18,19] have been characterized in tea flowers.

In our previous studies [17,18,19,20], tea flowers were found to contain a high amount of 1PE, which can be used as a fragrance in the food flavoring and cosmetic industries and as an intermediate in the pharmaceutical industry. However, it is unfeasible to extract and purify 1PE from tea flowers because doing so comes at a high cost, is time consuming, and produces a low yield. We have demonstrated that tea flowers contain enzymes that can transform from acetophenone to 1PE [17,20]. The cost of the substrate acetophenone is much lower than the cost of 1PE. In the present study, we established a synthetic system of 1PE production using acetophenone as a substrate and enzymes from tea flowers, and optimized the production conditions of 1PE using response surface methodology.

## 2. Results and Discussion

**Figure 1:**
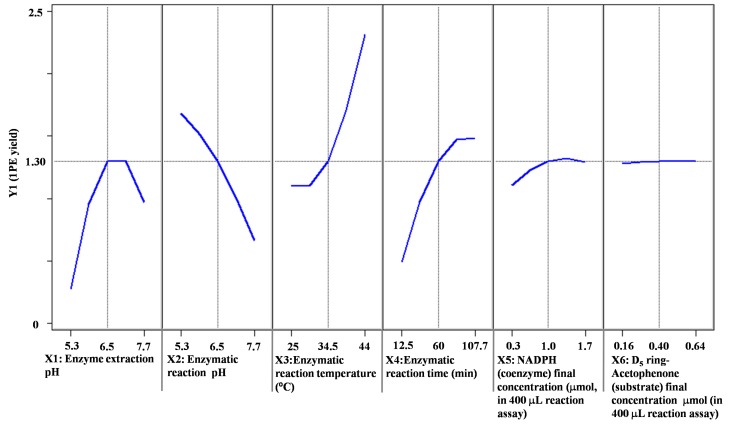
Prediction profilers of the effects of six variables (X1–X6) on 1PE yield. 1PE yield was determined by the peak area ratio of [^2^H_5_]ring-1PE to an internal standard (ethyl *n*-decanoate). Peak areas of the internal standard were calculated as the summation of *m*/*z* 88 and *m*/*z* 101. [^2^H_5_]ring-1PE peak areas were calculated as the summation of *m*/*z* 84, *m*/*z* 112, and *m*/*z* 127.

**Figure 2:**
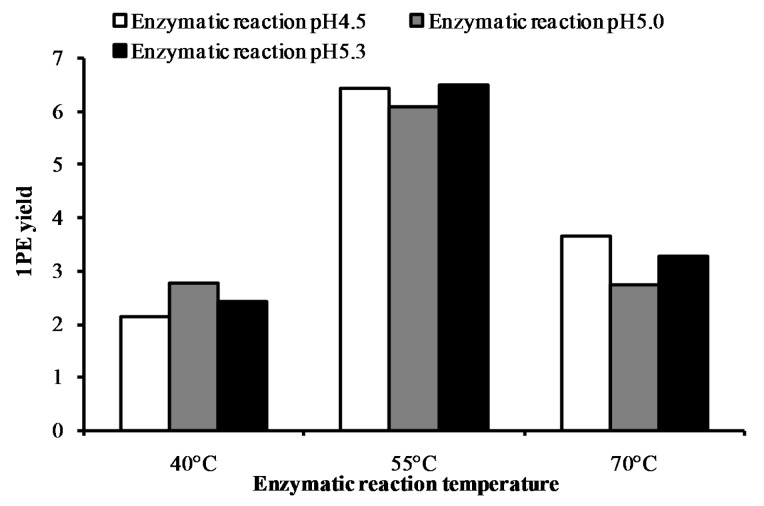
Effects of the enzymatic reaction pH (4.5–5.3) and the enzymatic reaction temperature (40–70 °C) on 1PE yield. 1PE yield was determined by the peak area ratio of [^2^H_5_]ring-1PE to an internal standard (ethyl *n*-decanoate). Peak areas of the internal standard were calculated as the summation of *m*/*z* 88 and *m*/*z* 101. [^2^H_5_]ring-1PE peak areas were calculated as the summation of *m*/*z* 84, *m*/*z* 112, and *m*/*z* 127.

**Figure 3:**
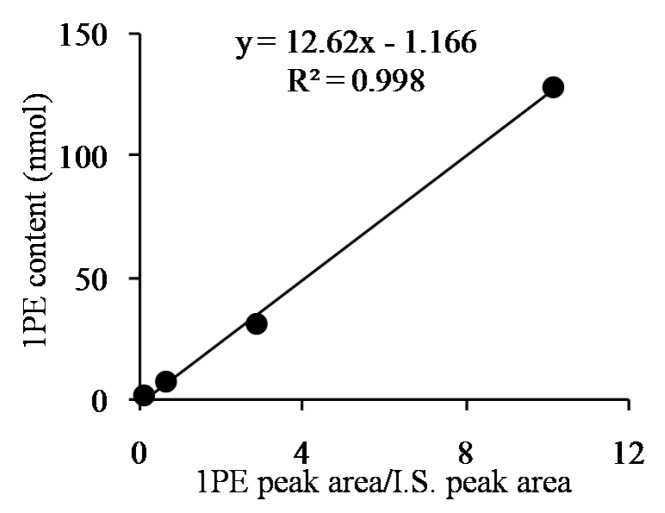
Standard 1PE curve using ethyl *n*-decanoate as an internal standard (IS). *X*-axis shows the GC-MS peak area ratio of 1PE to the IS. *Y*-axis shows 1PE content. The IS content was 5 nmol.

**Figure 4:**
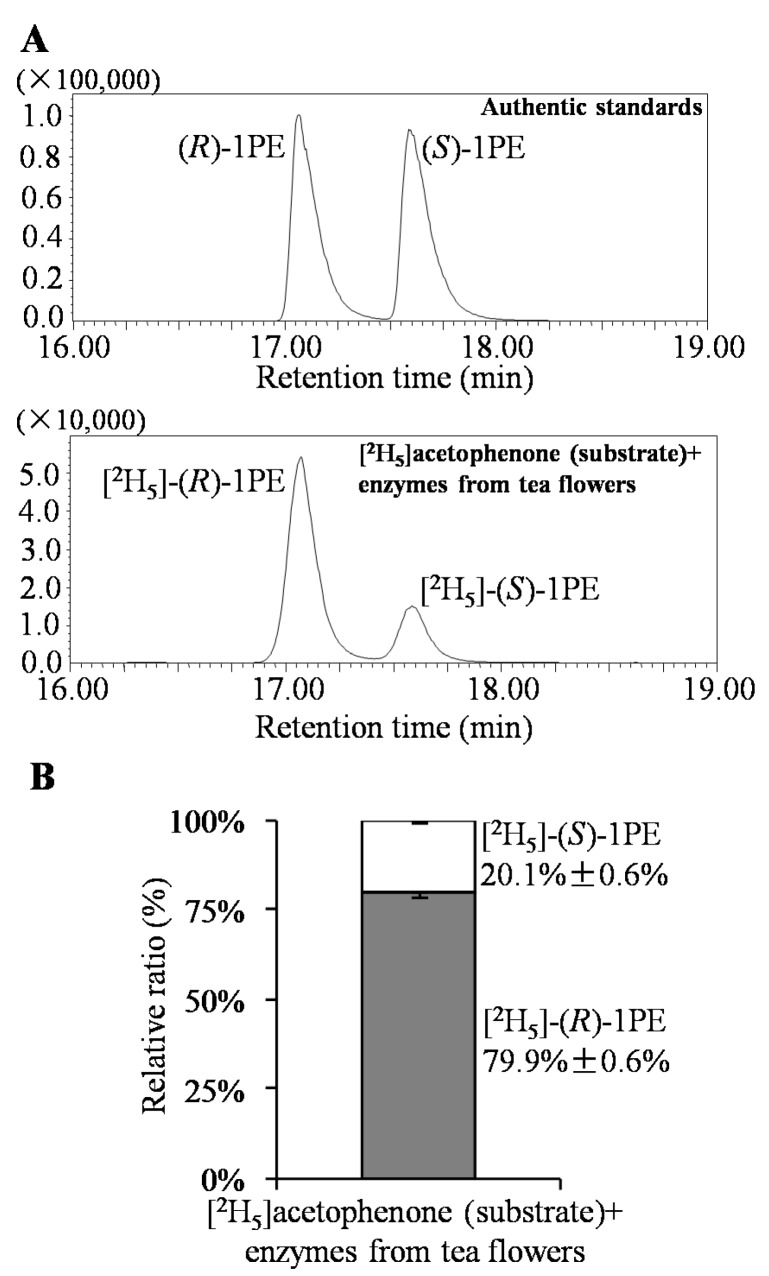
Identification of (*R*)-1PE and (*S*)-1PE from biotransformation using enzymes from tea flowers. (**A**) GC-MS equipped with an InertCap CHIRAMIX column was employed to determine (*R*)-1PE and (*S*)-1PE; (**B**) Data are expressed as mean ± S.D. (*n* = 5).

Response surface methodology with six factors (extraction pH, reaction pH, reaction temperature, reaction time, coenzyme NADPH concentration, and substrate acetophenone concentration) at five levels was employed to examine the effects of these parameters on 1PE yield (Table 1). There were 58 runs in the design and response value was Y1 (1PE production) (Table 2). Using response surface methodology, the four factors were optimized to acquire the highest 1PE yield with an enzyme extraction pH of 7.0, an enzyme reaction time of 100 min, a coenzyme NADPH concentration of 3.75 μmol/mL in the reaction assay (1.5 μmol in a 400 μL reaction assay), and a substrate acetophenone concentration of 1.25 μmol/mL in the reaction assay (0.5 μmol in a 400 μL reaction assay) (Figure 1). However, the other two factors including reaction pH and reaction temperature need to be further optimized. Therefore, the reaction pH ranging from 4.5 to 5.3 and the reaction temperature ranging from 40 °C to 70 °C were further investigated. The results showed that the highest 1PE yield was obtained under the conditions of a reaction pH of 5.3 and a reaction temperature of 55 °C (Figure 2). Based on calculations of the standard 1PE curve (Figure 3), the 80.8 nmol of 1PE was produced from 500 nmol of acetophenone in the optimized condition. Furthermore, the enzymes from tea flowers mainly produced (*R*)-1PE from acetophenone (Figure 4). In future industrial applications, considering cost, the additional amount of acetophenone can be reduced, because the substrate amount did not significantly affect 1PE yield (Figure 1). In addition, due to its relatively low boiling point, acetophenone may be easily released under a relatively high reaction temperature (55 °C) [17]. Further study on the improvement of the complete utilization of acetophenone as a substrate will be helpful for increases in yield and reductions in cost in industrial applications.

1PE can be used as a fragrance in the food flavoring and cosmetic industries and as an intermediate in the pharmaceutical industry. Some microorganisms including the *Azoarcus* sp. strain EbN1 and *Geotrichum candidum* NBRC 4597 were found to contain enzymes being able to convert acetophenone to 1PE [22,23]. However, such enzymes were not characterized in plants. In our previous study, using traditional protein chromatography, the two types of partially purified enzymes were proposed to be responsible for formations of (*R*)-1PE and (*S*)-1PE from acetophenone, respectively [20]. Furthermore, the major 1PE synthetic enzymes were to produce the (*R*)-1PE of tea flowers in vivo [17]. The present study characterized the in vitro properties of 1PE synthetic enzymes from tea flowers, which showed a higher activity under acidic condition and a higher temperature (Figure 1 and Figure 2). In addition, a medium pH extraction buffer was used to obtain 1PE synthetic enzymes with high activity (Figure 1).

**Figure 5:**
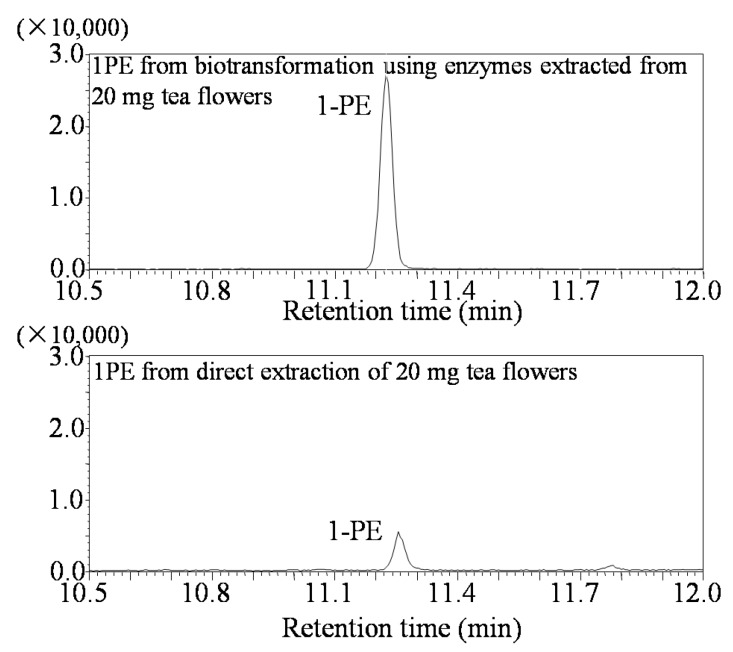
GC-MS identification of 1PE products from biotransformation using enzymes from tea flowers and direct extraction of tea flowers.

Although 1PE was accumulated in tea flowers in contrast to other *Camellia* plants [17], it is unfeasible to extract and isolate “natural 1PE” directly from tea flowers because doing so has a high cost, is time consuming, and produces a low yield. Therefore, 1PE synthetic enzymes in tea flowers can be used to produce “natural 1PE” in vitro. Figure 5 shows that 1PE production using enzymes from tea flowers was significantly higher than that from tea flowers (Figure 5). It is important to improve the performance of the enzyme synthesis systems and to increase the yield of the target product without increasing the cost. Response surface methodology is a combination of statistical and mathematical techniques by which a response is affected by several variables, and the aim is to optimize this response [24]. In recent years, response surface methodology has been widely applied to the optimization of biochemical processes, such as the enzymatic synthesis of fatty esters [25], alkaline protease production from *Bacillus mojavensis* in a bioreactor [26], the synthesis of butylgalactoside using β-galactosidase from *Aspergillus oryzae* [27], bioconversion from 2-phenylethanol to phenylacetaldehyde in a two-phase fed-batch system [28], the production of cholesterol oxidase using *Rhodococcusequi* no. 23 [29], and the production of phytase by *Pichia anomala* [30]. Response surface methodology was employed not only for the optimization of production but also for the determination of kinetic constants, enzyme stability, and kinetics [31]. In the present study, response surface methodology was used to optimize 1PE production using plant-derived enzymes. As there were six variables from extraction and reaction processes that possibly influenced 1PE yield (Table 1), the use of response surface methodology could reduce experimental runs from multiple variables and quickly obtain optimized conditions (Figure 1 and Figure 2). This study provided a good example of the optimization of functional metabolite production using plant-derived enzymes.

## 3. Materials and Methods

### 3.1. Extraction of Crude 1PE Synthetic Enzymes from Tea Flowers

Tea flowers (*C. sinensis* var. Yabukita) at stage-3 (the flower is fully open) contain a higher amount of 1PE [17], and these tea flowers were used to extract crude 1PE synthetic enzymes. One gram of finely powdered tea flowers at stage-3 crushed by a Multi-Beads Shocker (2000 rpm, 15 s) were added to 0.3 g of polyvinylpolypyrrolidone and 20 mg of 3-[(3-cholamidopropyl)-dimethylamino]-1-propanesulfonate, homogenized in 10 mL of cold buffer A (100 mM potassium phosphate buffer containing 1% glycerol and 1 mM ethylenediamine tetraacetic acid (EDTA)) under ice, and centrifuged (26,740× *g*, 4 °C, 20 min). The supernatant was centrifuged again (26,740× *g*, 4 °C, 20 min) to remove suspended substances, then loaded on a PD-10 desalting column (GE Healthcare Bio-Sciences, Piscataway, NJ, USA), and eluted using 10 mM potassium phosphate buffer containing 0.1% glycerol and 0.1 mM EDTA. The eluate was used as a crude enzyme solution.

### 3.2. The Optimization of Production Conditions of 1PE Using Crude 1PE Synthetic Enzymes from Tea Flowers

To exclude the possible effects from acetophenone and 1PE that occur in a crude enzyme solution, the substrate in the enzyme assay used labeled [^2^H_5_]ring-acetophenone (98%, Cambridge Isotope Laboratories, Inc., Andover, MA, USA). The reaction mixture contained 200 μL of enzyme solution, 50 μL of substrate (labeled [^2^H_5_]ring-acetophenone), 50 μL of coenzyme (NADPH), and 100 μL of a 100 mM potassium phosphate buffer. The different reaction conditions (Table 1) were investigated. Afterwards, 5 nmol of ethyl *n*-decanoate as an internal standard was added. The reaction products were extracted with 0.4 mL of hexane/ethyl acetate (1:1) and centrifuged (10,000× *g*, 4 °C, 3 min). The supernatant was dried over anhydrous sodium sulfate. Samples were then analyzed by GC-MS, which was same as the previous study [20]. The temperature of the injector was 230 °C. The GC was equipped with a capillary SUPELCOWAX™ 10 column (Supelco Inc., Bellefonte, PA, USA, 30 m × 0.25 mm I.D., 0.25 μm film thickness). Helium was used as a carrier gas at a flow rate of 1.6 mL/min. The GC oven was maintained at 60 °C for 3 min. The temperature of the oven was programmed at 40 °C/min to 180 °C and then at 10 °C/min to 240 °C, and kept at this temperature for 3 min. The mass spectrometry was operated by the full scan mode (mass range *m*/*z* 70–200). Characteristic ions of [^2^H_5_]ring-1PE were *m*/*z* 84, *m*/*z* 112, and *m*/*z* 127.

### 3.3. Analysis of (R)-1PE and (S)-1PE Products from Biotransformation Using Enzymes from Tea Flowers

GC-MS equipped with an InertCap CHIRAMIX column (30 m × 0.25 mm × 0.25 μm, GL Sciences, Inc., Torrence, CA, USA) was employed to determine (*R*)-1PE and (*S*)-1PE. The injector temperature was 180 °C, splitless mode was used with a splitless time of 1 min, and helium was the carrier gas with a velocity of 1.0 mL/min. The GC temperatures were as follows: 60 °C for 2 min, a ramp of 40 °C/min to 105 °C, followed by 2 °C/min to 137 °C, then 80 °C/min to 180 °C, and 180 °C for 10 min.

### 3.4. Statistical Analysis

Response surface methodology was processed using the software package SAS v8.0 (SAS Institute Inc., Cary, NC, USA).

## 4. Conclusions

Our present study characterized the in vitro properties of 1PE synthetic enzymes from tea flowers, suggesting that the enzymes were thermostable, and the enzyme extraction pH and the reaction pH were different at the highest enzyme activity. Furthermore, this study established an effective and low-cost system for producing 1PE, with the following optimized conditions: an extraction pH of 7.0, a reaction pH of 5.3, a reaction temperature of 55 °C, a reaction time of 100 min, a coenzyme NADPH concentration of 3.75 μmol/mL in the reaction assay, and a substrate acetophenone concentration of 1.25 μmol/mL in the reaction assay. These results provide essential information for future industrial 1PE production using plant-derived enzymes. In addition, this will contribute to future applications of functional proteins from abundant and waste tea flower resources.

## Figures and Tables

**Table 1:** Factors and levels of response surface methodology.

Factors (Coded Level)	−2.37841	−1	0	1	2.37841
X1: Enzyme extraction pH	5.3	6	6.5	7	7.7
X2: Enzymatic reaction pH	5.3	6	6.5	7	7.7
X3: Enzymatic reaction temperature (°C)	25	30	34	38	44
X4: Enzymatic reaction time (min)	12.5	40	60	80	107.5
X5: NADPH (coenzyme) final concentration μmol (in 400 μL reaction assay)	0.3	0.7	1	1.3	1.7
X6: [^2^H_5_]ring-acetophenone (substrate) final concentration μmol (in 400 μL reaction assay)	0.16	0.3	0.4	0.5	0.64

**Table 2:** Response surface methodology for different conditions of 1PE production and 1PE yield.

Run	X1	X2	X3	X4	X5	X6	Y1 ^a^ (1PE Yield)
1	−1	−1	−1	−1	−1	−1	0.758559371
2	−1	−1	−1	−1	1	1	0.846748345
3	−1	−1	−1	1	−1	1	1.153593462
4	−1	−1	−1	1	1	−1	1.150490973
5	−1	−1	1	−1	−1	1	1.025881547
6	−1	−1	1	−1	1	−1	1.154783234
7	−1	−1	1	1	−1	−1	1.586798695
8	−1	−1	1	1	1	1	1.452172855
9	−1	1	−1	−1	−1	1	0.447787063
10	−1	1	−1	−1	1	−1	0.508368068
11	−1	1	−1	1	−1	−1	0.765378069
12	−1	1	−1	1	1	1	0.631801149
13	−1	1	1	−1	−1	−1	0.620733457
14	−1	1	1	−1	1	1	0.770290718
15	−1	1	1	1	−1	1	1.05186958
16	−1	1	1	1	1	−1	1.269535781
17	1	−1	−1	−1	−1	1	0.828065574
18	1	−1	−1	−1	1	−1	0.953103788
19	1	−1	−1	1	−1	−1	1.110510204
20	1	−1	−1	1	1	1	2.091175436
21	1	−1	1	−1	−1	−1	1.490051857
22	1	−1	1	−1	1	1	1.525848802
23	1	−1	1	1	−1	1	1.838977526
24	1	−1	1	1	1	−1	2.564189622
25	1	1	−1	−1	−1	−1	0.652233718
26	1	1	−1	−1	1	1	0.782094508
27	1	1	−1	1	−1	1	0.937807534
28	1	1	−1	1	1	−1	1.035549206
29	1	1	1	−1	−1	1	1.105724121
30	1	1	1	−1	1	−1	1.009265424
31	1	1	1	1	−1	−1	1.500473779
32	1	1	1	1	1	1	1.35519365
33	−2.37841	0	0	0	0	0	0.481731606
34	2.378414	0	0	0	0	0	0.698984848
35	0	−2.37841	0	0	0	0	1.570230689
36	0	2.378414	0	0	0	0	0.722668367
37	0	0	−2.37841	0	0	0	0.896550121
38	0	0	2.378414	0	0	0	2.489739273
39	0	0	0	−2.37841	0	0	0.548374842
40	0	0	0	2.378414	0	0	1.370597491
41	0	0	0	0	−2.37841	0	1.263502722
42	0	0	0	0	2.378414	0	1.074987099
43	0	0	0	0	0	−2.37841	1.103270031
44	0	0	0	0	0	2.378414	1.421635721
45	0	0	0	0	0	0	1.226729653
46	0	0	0	0	0	0	1.377231877
47	0	0	0	0	0	0	1.242217123
48	0	0	0	0	0	0	1.291999792
49	0	0	0	0	0	0	1.273597247
50	0	0	0	0	0	0	1.249548763
51	0	0	0	0	0	0	1.224737373
52	0	0	0	0	0	0	1.28414853
53	0	0	0	0	0	0	1.263331053
54	0	0	0	0	0	0	1.250814756
55	0	0	0	0	0	0	1.216741671
56	0	0	0	0	0	0	1.308596416
57	0	0	0	0	0	0	1.225345486
58	0	0	0	0	0	0	1.360042105

^a^ The formed [^2^H_5_]ring-1PE amount (1PE yield) was determined by the peak area ratio of the analyte to an internal standard (ethyl *n*-decanoate). Peak areas of the internal standard were calculated as the summation of *m*/*z* 88 and *m*/*z* 101. [^2^H_5_]ring-1PE peak areas were calculated as the summation of *m*/*z* 84, *m*/*z* 112, and *m*/*z* 127.
